# Charcot Arthropathy of the Shoulder With Ipsilateral Wrist and First Carpometacarpal Joint Involvement Secondary to Chiari Malformation Type I With Syringomyelia

**DOI:** 10.7759/cureus.112565

**Published:** 2026-07-13

**Authors:** Ali Alsuwaidi, Radjah Farhi, Ahmed Fouad Ezzeldein Abdalrahman, Olivier Verborgt, Aisha Wasi

**Affiliations:** 1 Orthopedics, Tarmeem Orthopedic and Spine Specialty Hospital, Abu Dhabi, ARE

**Keywords:** arnold-chiari malformation, arthropathy, carpometacarpal joints, neurogenic, shoulder joint, syringomyelia, wrist joint

## Abstract

Charcot arthropathy of the shoulder is a rare manifestation of neuropathic joint disease and is most commonly associated with syringomyelia. When present, it often results in progressive joint destruction, but, due to minimal pain, it leads to delayed diagnosis. We report the case of a 54-year-old female presenting with a three-month history of left shoulder swelling, deformity, and severe restriction of range of motion, associated with ipsilateral wrist deformity. The patient had a known history of Chiari malformation type I with syringomyelia. Clinical examination revealed marked shoulder deformity with severe restriction of active motion and reduced sensation in the affected limb. Radiographs demonstrated advanced destructive changes of the glenohumeral joint with humeral head deformity and loose bodies. MRI revealed extensive shoulder joint effusion, rotator cuff muscle atrophy with relative deltoid preservation, and an extensive cervical syrinx extending from C2 to C7. A diagnosis of neuropathic (Charcot) arthropathy of the shoulder secondary to Chiari malformation with syringomyelia was established. Additional imaging showed obliteration of the first carpometacarpal (CMC) joint with subchondral sclerosis, cystic changes, and volar subluxation of the first metacarpal, as well as early degenerative changes at the ipsilateral elbow. The patient was managed conservatively with analgesics, protective measures, and multidisciplinary follow-up. Charcot arthropathy of the shoulder is an uncommon but important complication of syringomyelia associated with Chiari malformation type I. This case highlights the importance of early recognition of neuropathic arthropathy in patients with neurological disease. The combined involvement of the shoulder, wrist, and first CMC joint represents an unusual pattern of upper-limb neuropathic arthropathy and contributes to the existing literature on multi-joint involvement.

## Introduction

Charcot shoulder is a neuropathic shoulder arthropathy. It is a pathological process (peripheral or central) leading to destruction of the joint. It is frequently diagnosed late, resulting in extensive bone and joint destruction. It most commonly affects the weight-bearing joints but may affect upper extremities in some cases [1,2]. The rare cases of upper joint extremities, such as shoulders, are associated with conditions related to the central nervous system, such as syringomyelia [1,3]. In contrast to Charcot arthropathy of the foot and ankle, which is commonly associated with diabetes mellitus, neuropathic arthropathy of the shoulder is more frequently linked to central neurological pathology, making neurological evaluation an important component of diagnosis and management [4,5]*. *Even more uncommonly, the distal joints of the ipsilateral limb, such as the wrist and the first carpometacarpal (CMC) joint, may be involved [6]. The association of syringomyelia with Chiari malformation type I leads to progressive cavitation of the spinal cord, resulting in dissociated sensory loss, proprioceptive impairment, and muscle atrophy [4]. The loss of nociceptive and thermal sensation predisposes affected joints to repetitive microtrauma, ultimately culminating in severe joint destruction [3,7,8]. Charcot shoulder is therefore an uncommon but well-recognized complication of long-standing syringomyelia and often presents with joint swelling, deformity, and profound functional limitation despite minimal pain [1,3]. This relative lack of pain despite severe radiological destruction is an important clinical clue and may contribute to delayed diagnosis. The diagnosis of Charcot shoulder is challenging due to its rarity and its ability to mimic other conditions, such as septic arthritis, inflammatory arthropathy, rotator cuff arthropathy, or neoplastic processes [2]. Radiographic and magnetic resonance imaging (MRI) findings typically reveal joint effusion, osseous fragmentation, articular surface destruction, and periarticular soft tissue changes [9]. Early recognition is essential to avoid unnecessary interventions and to guide appropriate management. Management of neuropathic shoulder arthropathy remains controversial, with treatment strategies ranging from conservative measures to surgical intervention in selected cases [10]. However, surgical outcomes are often unpredictable due to ongoing neurological impairment and a high risk of prosthetic failure. Surgical outcomes are also unpredictable because persistent neurological impairment, poor bone stock, instability, and loss of protective sensation may predispose the joint to recurrent destruction, prosthetic loosening, and implant failure [9]. Consequently, individualized, multidisciplinary management is frequently recommended. This includes orthopedic input for surgical decision-making and complication management, neurological evaluation to assess the extent and progression of neuromuscular involvement, structured physiotherapy to improve range of motion and strength, and comprehensive rehabilitation programs to enhance functional independence. In some cases, additional input from pain specialists and occupational therapists may further optimize patient-centered outcomes [11].

This case report describes a rare presentation of Charcot arthropathy of the shoulder secondary to Chiari malformation type I with syringomyelia, accompanied by ipsilateral wrist deformity and first CMC joint involvement, which is an extremely uncommon presentation. The report highlights the clinical presentation, radiological features, diagnostic challenges, and management considerations, aiming to contribute to the limited literature on this uncommon condition. This case is clinically important because delayed recognition of neuropathic arthropathy may lead to unnecessary investigations or interventions, while early diagnosis can guide appropriate imaging, neurological assessment, joint protection, and multidisciplinary management.

## Case presentation

A 54-year-old female presented with a three-month history of left shoulder swelling and severe restriction of range of motion, associated with ipsilateral left-hand deformity. There was no history of significant trauma preceding the onset of symptoms. The patient reported functional limitation rather than pain as the primary concern. The patient was a known case of Chiari malformation type I with syringomyelia, diagnosed previously and managed conservatively. No acute neurological deterioration was reported at the time of presentation. The clinical course, investigations, diagnosis, and management are summarized in Table 1.

**Table 1:** Patient timeline.

Time point	Event/Finding
Previous history	Known Chiari malformation type I with syringomyelia, managed conservatively
3 months before presentation	Onset of left shoulder swelling, deformity, and restricted movement
At presentation	Minimal pain, marked shoulder effusion, severe restriction of active range of motion, and reduced sensation
Initial investigations	Laboratory investigations showed mildly elevated ESR, CRP 3.4 mg/L, anemia, and normal leukocyte and platelet counts Radiographs and MRI showed destructive shoulder changes, ipsilateral wrist/first CMC involvement, and cervical syrinx
Diagnosis	Charcot arthropathy of the left shoulder secondary to Chiari malformation with syringomyelia
Management and follow-up	Conservative treatment, protective measures, multidisciplinary follow-up; stable status with reduced pain

Clinical examination

Upon examination, the left shoulder showed marked joint effusion. The joint was not painful on palpation. Active range of motion was severely restricted, with forward elevation of less than 10°, external rotation of 0°, internal rotation of 0°, and abduction of 0°. Passive range of motion showed partial preservation, with forward elevation up to 110°, while external and internal rotation remained absent. A formal visual analog scale pain score was not available retrospectively.

Muscle power testing revealed deltoid strength of 4/5. Sensory examination of the left upper limb demonstrated diminished sensation and temperature compared to the contralateral side. Formal dermatome-based sensory mapping was not available retrospectively.

Examination of the ipsilateral elbow showed no obvious deformity. Examination of the left wrist revealed ulnar deviation with first CMC joint effusion, tender to palpation with restricted range of motion, and decreased temperature sensation compared to the contralateral side.

Laboratory evaluation demonstrated a mildly elevated erythrocyte sedimentation rate (ESR), with a normal C-reactive protein (CRP) level. The ESR was 32 mm/hr, and the CRP was 3.4 mg/L. Complete blood count revealed anaemia, with hemoglobin of 10.0 g/dL, hematocrit of 31.4%, and red blood cell count of 3.71 × 10⁶/µL, while total leukocyte and platelet counts were within normal limits. Reticulocyte parameters obtained during the initial assessment were also within normal limits. Routine electrolyte and renal function parameters were unremarkable. The relevant laboratory findings are summarized (Table 2).

**Table 2:** Laboratory findings demonstrated a mildly elevated ESR with a normal CRP level, anemia reflected by low hemoglobin, hematocrit, and red blood cell count, and normal reticulocyte parameters. Total leukocyte and platelet counts were within normal limits.

Time point	Laboratory parameter	Patient value	Reference range	Interpretation
At presentation	Erythrocyte sedimentation rate (ESR)	32 mm/hr	0-20 mm/hr	Mildly elevated
At presentation	C-reactive protein (CRP)	3.4 mg/L	0.0-5.0 mg/L	Within normal range
At presentation	Hemoglobin	10.0 g/dL	11.7-16.0 g/dL	Low
At presentation	Hematocrit	31.40%	35-47%	Low
At presentation	Red blood cell count	3.71 × 10⁶/µL	3.8-5.2 × 10⁶/µL	Low
Initial assessment	Reticulocyte count	46.10 × 10⁹/L	25.0-75.0 × 10⁹/L	Within normal range
Initial assessment	Reticulocyte percentage	1.20%	0.5-1.6%	Within normal range
Initial assessment	Reticulocyte hemoglobin equivalent	32.4 pg	29.8-37.7 pg	Within normal range
At presentation	Total leukocyte count	Within normal limits	4.0-11.0 × 10⁹/L	Normal
At presentation	Platelet count	Within normal limits	150-400 × 10⁹/L	Normal

Radiological assessment

**Figure 1:**
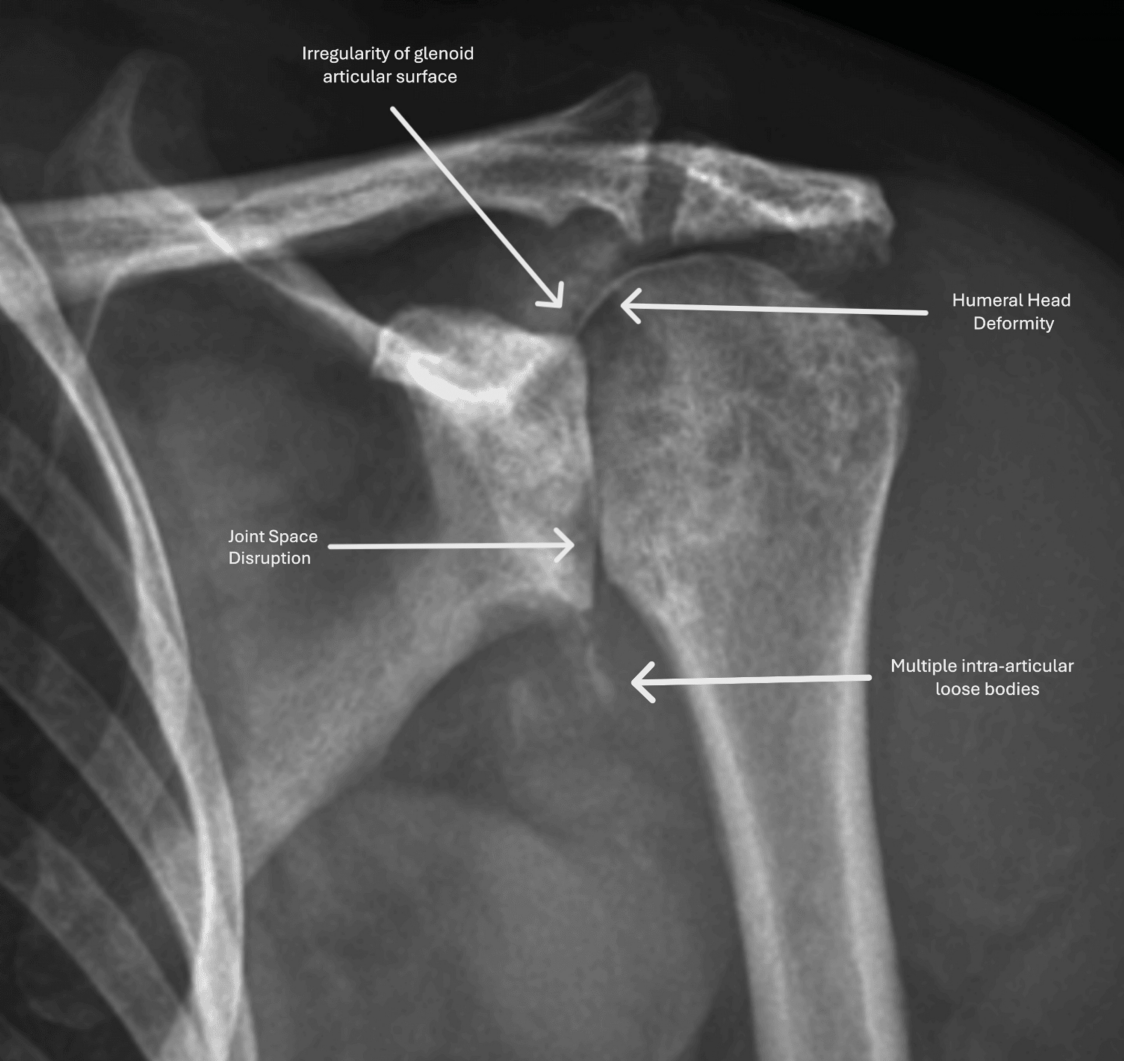
External rotation radiograph of the left shoulder demonstrating advanced destructive changes of the glenohumeral joint, including humeral head deformity, irregularity of the glenoid articular surface, joint space disruption, and multiple intra-articular loose bodies, consistent with Charcot neuropathic arthropathy.

**Figure 2:**
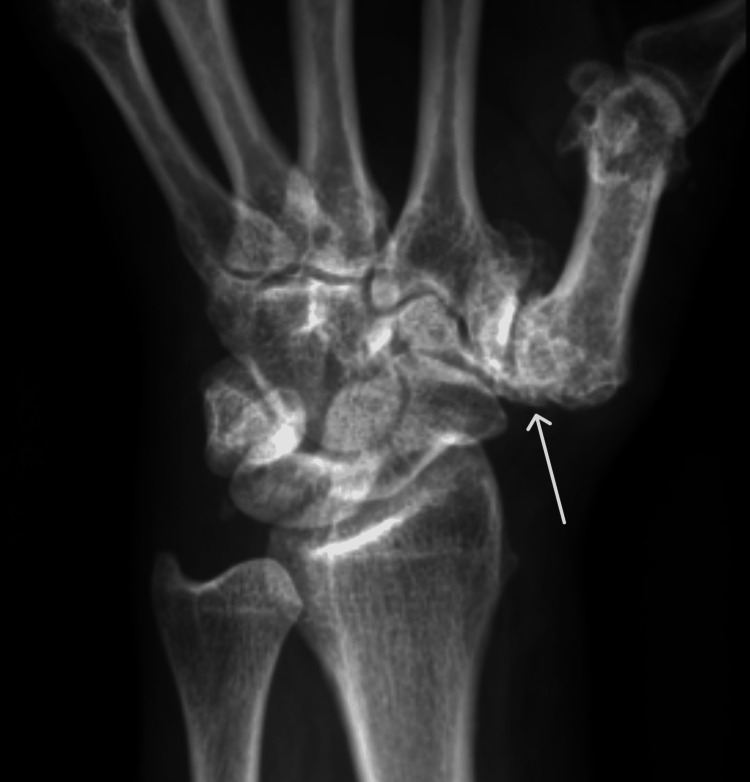
Oblique radiograph of the left wrist demonstrating advanced neuropathic arthropathic changes, including joint space obliteration, subchondral sclerosis, cystic changes, bony fragmentation, carpal disorganization, and volar subluxation of the first metacarpal, consistent with ipsilateral wrist and first carpometacarpal joint involvement secondary to syringomyelia. The abnormalities are indicated by white arrows.

**Figure 3:**
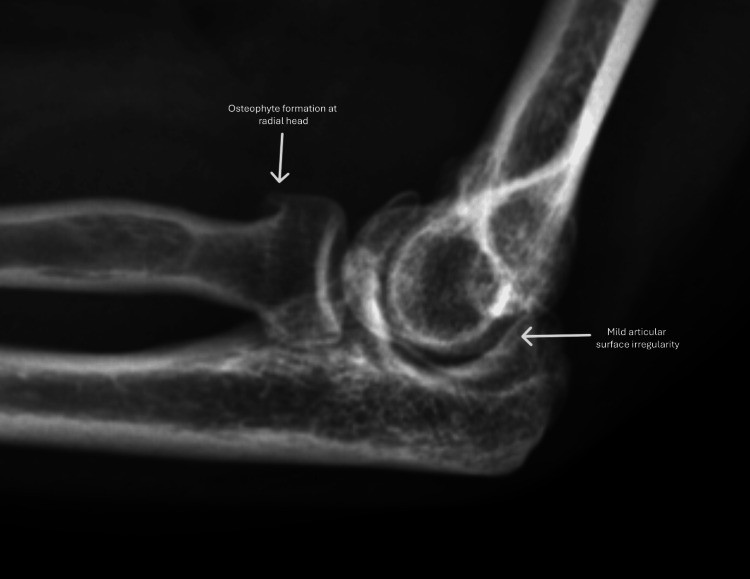
Lateral radiograph of the left elbow demonstrating early degenerative and neuropathic changes, including radial head osteophyte formation and mild articular surface irregularity, suggestive of early ipsilateral joint involvement.

Plain radiographs of the left shoulder showed severe joint destruction, deformity, and multiple loose bodies inferior to the glenoid (Figure 1). Distal joint involvement was also evident, with obliteration of the first CMC joint, subchondral sclerosis, cystic changes, and volar subluxation of the first metacarpal (Figure 2). Early osteophytic changes were additionally noted at the ipsilateral radial head (Figure 3).

**Figure 4:**
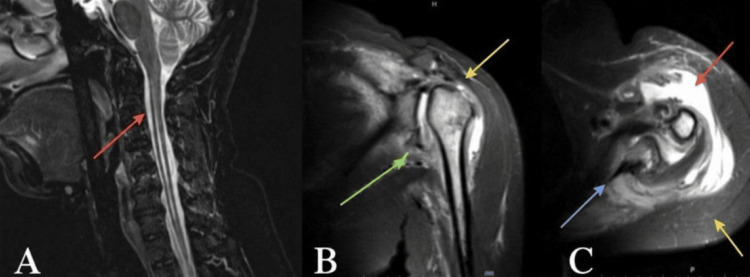
Magnetic resonance imaging findings. (A) Sagittal cervical spine MRI demonstrating an extensive syrinx extending from C2 to C7 in the setting of Chiari I malformation. (B) Coronal oblique shoulder MRI showing humeral head deformity, glenoid erosion, and marked glenohumeral joint effusion. (C) Axial shoulder MRI demonstrating extensive joint and periarticular fluid with rotator cuff muscle atrophy and relative preservation of the deltoid, consistent with neuropathic arthropathy.

MRI of the left shoulder demonstrated extensive joint effusion involving the glenohumeral, subacromial, and subdeltoid spaces, along with marked rotator cuff muscle atrophy with relative preservation of the deltoid muscle (Figures 4B-4C). Humeral head flattening with associated glenoid erosions was also present.

Cervical spine MRI demonstrated signs of syringomyelia with an extensive syrinx extending from C2 to C7 (Figure 4A). 

Diagnosis

Based on the clinical presentation, neurological history, and radiological findings, a diagnosis of neuropathic arthropathy (Charcot joint) of the left shoulder secondary to Chiari malformation type I with syringomyelia was established, with early involvement of the ipsilateral wrist and first CMC joint.

Management

The patient was managed conservatively. She was prescribed etoricoxib 90 mg once daily for 10 days for symptomatic relief and advised to continue protective and supportive measures for the affected limb. Given the complexity of the condition, a referral was made for multidisciplinary evaluation involving orthopedic, neurological, and physiotherapy teams to guide further management. The patient was followed up on every three months. At the most recent follow-up, the clinical status remained stable with reduced pain.

## Discussion

Neuropathic arthropathy, also known as Charcot joint, is a chronic, progressive degenerative condition resulting from impaired sensory innervation, leading to progressive joint destruction. Although most commonly associated with diabetes and affecting weight-bearing joints, involvement of the upper extremity is uncommon and is most frequently linked to syringomyelia [1,2]. A summary of previously reported cases of syringomyelia-associated upper-limb neuropathic arthropathy is provided (Table 3). Syringomyelia is characterized by cavitation of the spinal cord, typically involving the cervical region, and is commonly associated with Chiari malformation type I. Disruption of the decussating fibres of the lateral spinothalamic tract results in loss of pain and temperature sensation while relatively preserving proprioception [10]. This sensory dissociation predisposes affected joints to repetitive microtrauma, leading to progressive structural joint damage characteristic of neuropathic arthropathy [3]. Unlike diabetic Charcot arthropathy, which classically affects the foot and ankle, syringomyelia-related neuropathic arthropathy of the upper limb should prompt careful neurological evaluation, as the musculoskeletal presentation may be the first indication of an underlying spinal cord pathology [5-7].

**Table 3:** Summary of previously reported cases of syringomyelia-associated upper limb neuropathic arthropathy.

Author/year	Underlying pathology	Joint involvement	Management	Clinical outcome/relevance
Kumar et al., 2011 [8]	Chiari I malformation with syringomyelia	Shoulder	Radiological diagnosis, followed by conservative/supportive management and neurological evaluation	Highlighted the role of MRI in demonstrating both destructive shoulder arthropathy and the underlying cervical syrinx
Grahovac et al., 2011 [10]	Chiari I malformation with syringomyelia	Shoulder	Conservative management with long-term clinical follow-up	Reported Charcot shoulder caused by Chiari I malformation with syringomyelia and emphasized the chronic progressive nature of the condition
Butala et al., 2014 [6]	Cervical syringomyelia	Ipsilateral shoulder and thumb carpometacarpal joint	Conservative/supportive management	Particularly relevant to the present case because it described combined ipsilateral shoulder and thumb CMC joint neuropathic arthropathy
Nambiar et al., 2018 [3]	Syringomyelia	Shoulder	Conservative management with neurological assessment	Reinforced syringomyelia as an important underlying cause of neuropathic shoulder arthropathy
Mahmoud et al., 2022 [1]	Chiari malformation with syringomyelia	Shoulder	Conservative/supportive treatment with multidisciplinary neurological and orthopedic follow-up; surgical intervention considered only in selected cases	Systematic literature review emphasizing delayed diagnosis and the importance of considering Chiari malformation with syringomyelia in destructive shoulder arthropathy
Shi et al., 2023 [11]	Cervical spondylotic myelopathy with syringomyelia	Multiple joints	Multidisciplinary management	Demonstrated that multi-joint Charcot arthropathy may occur in association with spinal cord pathology and syringomyelia
Arabzadeh et al., 2025 [2]	Arnold-Chiari malformation with syringomyelia	Shoulder	Clinical and radiological assessment followed by case-based management; conservative treatment emphasized	Recent case report supporting the association between Arnold-Chiari malformation, syringomyelia, and Charcot arthropathy

Clinically, patients typically present with painless swelling, progressive deformity, and severe functional limitation despite advanced joint destruction. Reduced range of motion and swelling are the most commonly reported presenting symptoms, while pain may be absent in a substantial proportion of cases [1]. This relative lack of pain often leads to delayed diagnosis and misdiagnosis as septic arthritis, inflammatory arthropathy, or neoplasm [2]. The discrepancy between the severity of radiological destruction and the relative absence of pain is an important diagnostic clue and should raise suspicion for neuropathic arthropathy, particularly in patients with known or suspected syringomyelia.

The shoulder and elbow are the most frequently affected joints in syringomyelia-related neuropathic arthropathy [6]. In contrast, distal joint involvement, such as the wrist and first carpometacarpal joint, is rarely described, making the present case particularly unusual and unique. Multi-joint involvement within the same limb has been reported but remains uncommon [12].

Additionally, very few reports describe involvement of the first carpometacarpal joint in association with Charcot shoulder. Previous case reports have described ipsilateral shoulder and thumb carpometacarpal joint neuropathic arthropathy, highlighting the exceptional rarity of this distribution pattern [7].

Radiographically, neuropathic arthropathy is characterized by joint destruction, bone resorption, fragmentation, and subluxation or dislocation. MRI plays a critical role in identifying both joint destruction and underlying neurological pathology, particularly spinal cord syrinx formation [9,13,14]. Therefore, when destructive shoulder arthropathy is identified without a clear traumatic, infectious, or inflammatory cause, MRI evaluation of the cervical spine should be considered to exclude syringomyelia or Chiari malformation-associated pathology [6,9,11,14].

The pathogenesis of neuropathic arthropathy remains multifactorial. Two primary mechanisms have been proposed: the neurotraumatic theory, which suggests repeated unrecognized trauma leads to joint destruction, and the neurovascular theory, which proposes autonomic dysfunction leading to hyperaemia and osteoclastic bone resorption [6,15,16].

Management remains challenging and controversial. Conservative treatment, including joint protection, immobilization, analgesia, and physiotherapy, is typically first-line. Surgical treatment may be considered in advanced cases but carries increased risk of failure due to persistent neurological impairment and poor bone quality [10,17]. Although shoulder arthroplasty has been described in selected patients with Charcot arthropathy, outcomes remain different, and careful patient selection is essential because instability, loosening, and prosthetic failure remain important concerns [10,15]. For this reason, treatment should be individualized according to pain severity, functional limitation, neurological status, bone stock, and patient expectations.

This case highlights the importance of considering neuropathic arthropathy in patients with known Chiari malformation and syringomyelia who present with progressive joint deformity, particularly when pain is minimal despite marked structural destruction. The associated involvement of the ipsilateral wrist and first carpometacarpal joint makes this presentation unusual and adds to the limited literature on multi-joint neuropathic arthropathy of the upper limb. Recognition of this pattern is clinically relevant because it may prompt appropriate neurological imaging, avoid misdiagnosis, and support early multidisciplinary management.

## Conclusions

Charcot arthropathy of the shoulder is an uncommon complication of syringomyelia associated with Chiari malformation type I. This diagnosis should be considered in patients presenting with minimally painful destructive shoulder arthropathy, sensory impairment, and progressive upper-limb deformity. The present case demonstrates an unusual pattern of ipsilateral shoulder, wrist, and first CMC joint involvement. Early recognition and appropriate imaging are important to guide management and limit further joint damage where possible.
